# Abstract of the Proceedings of the Buffalo Medical Association,
Reported by the Secretary

**Published:** 1859-04

**Authors:** Benj. H. Lemon


ART. III.—*Abstract of Proceedings of the Buffalo Medical Association.*

TUESDAY EVENING, March 1, 1859.

The Association met.

Present—The President, Dr. Wyckoff in the chair, and Drs. Wilcox, Eastman, Strong, Hawley, Butler, Gay, Ring, Treat, Lemon, Hutchins, Newman, and Whitney.

The secretary being absent, Dr. Lemon was appointed secretary *pro tem.*

Dr. GOULD reported the following case:

January 9, was called to see Mr. A. W., aged 33 years; about a year ago was sick with typhus fever, and had not been well since. Had been treated for debility last fall; complained at that time of lassitude and want of energy; said his complaint was laziness, as he suffered no pain; appetite good, slept well, &c. January 5th, he was advised to take a lobelia emetic, which operated powerfully. From that time to the 9th he grew gradually deaf, and at the time I visited him, he could scarcely be made to hear anything. This continued until the 16th, when diarrhœa set in, and patient died on the 17th. I have omitted the treatment, which consisted of light alteratives, blisters, applications of iodine, &c., as the indications required.

*Post-mortem*, sixteen hours after death. Present Drs. Wilcox, Lemon, Gould, and medical students Gould and Robinson. There was some congestion of the brain. Between the membranes and surface, several small clots were found, and some slight adhesions. The blood appeared like a mixture of pus and blood; corpus callosum softened; effusion in right venticle; choroid plexus injected; whole brain appeared injected and softened. On opening abdomen, found the liver and spleen enormously enlarged. The liver weighed twelve pounds and twelve ounces; the spleen six pounds and twelve ounces; the stomach and intestines healthy; kidneys same; about four ounces of fluid in the pericardium; all other organs healthy. The blood in all parts of the body had the same dark, grumous, and partially decomposed appearance, as noticed in the brain, and presenting an unusual appearance not easily explained.

The liver exceeded the usual weight, by at least eight and a half or nine pounds, and the spleen by at least six pounds.

Specimens of each were furnished Dr. Austin Flint, jr., for microscopical examination.*

Dr. Wyckoff exhibited to the Association, specimens of tape-worm, and gave the following history of the case:

February 12, 1859, Mrs. A., aged 27 years, called upon me with a piece, or rather four pieces, of tænia solium, in all about two yards.

Mrs. A. says she has enjoyed uniformly good health until the last seven or eight months, during which time she has complained of a gnawing pain in the epigastrium, sometimes a sensation of sinking, general lassitude, pain in the limbs, dimness of vision, dizziness, a dull heavy headache, loss of appetite, a dry cough or sensation of choking, difficulty of swallowing, sore throat, and she thought she felt something in the œsophagus, as if crawling or moving up and down.

I prescribed half a pint of dried pumpkin seeds, well bruised, made into a decoction with a quart of water; eight ounces to be taken every four hours; the first in the morning, fasting, and the whole followed by oleum ricini ℥, which operated upon the bowels three times. The first two dejections contained a few small pieces of the tænia, intermixed with fœcal matter; but at the third, the whole mass of worms was expelled, entirely free from fœcal matter.

Upon examination, assisted by Dr. Miner, I find the small tapering portion near the head of five different worms, and the head of none. In measuring all the pieces carefully, we found the whole amounting to forty-one yards, the longest worm about seven yards.

Two days after the expulsion of this large mass of worms, I repeated the dose of pumpkin seeds, at the urgent request of my patient, but without effect.

---

*These specimens were examined, but not very minutely; no changes in their intimate structure were discovered.—A. F., Jr.

November, 1856, the former dose was repeated. Since that time the patient has had uniform good health.

Dr. TREAT had observed a species of tape-worm in the "blue pike," which is caught in Lake Erie. He had formerly used pumpkin seed infusion, when it had such a reputation in this disease. He had used granulated tin, with success in two cases, thirty feet having passed at one evacuation in one case, and about the same quantity in the other.

A discussion then followed among the members of the society, on the different varieties of tape-worm.

Dr. TREAT made some remarks, which gave rise to a discussion, on the propriety of great caution, on the part of medical men, in giving the sanction of their names to instruments.

The Association then adjourned.

BENJ. H. LEMON, M. D.,
Secretary *pro tem.*

